# Reproductive plans and knowledge of assisted reproductive techniques among lesbian women: an international survey study

**DOI:** 10.5935/1518-0557.20230013

**Published:** 2023

**Authors:** Pedro Brandão, Rafal Zadykowicz, Rebecca Miscioscia, António de Pinho, Maria Liz-Coelho, Lavinia Iftene, Anita Ungure, Nathan Ceschin

**Affiliations:** 1 Ginemed Porto, Porto, Portugal; 2 Faculty of Medicine, University of Porto, Porto, Portugal; 3 Clinical Department of Obstetrics, Perinatology and Gynaecology, Medical University of Warsaw, Warsaw, Poland; 4 Fertility Center Aarhus, Aarhus, Denmark; 5 Department of Obstetrics and Gynecology, Centro Hospitalar Tâmega e Sousa, Penafiel, Portugal; 6 Clinical Hospital Nicolae Malaxa, Bucharest, Romania; 7 Riga Stradins University, Riga, Latvia; 8 Department of Emergency Care, Riga East Clinical University, Riga, Latvia; 9 Department of Gynaecology and Obstetrics, Ogre’s Regional Hospital, Ogre, Latvia; 10 Felicittá Fertility Institute, Curitiba, Brazil

**Keywords:** adoption, assisted reproduction technology, IVF, lesbian, reproduction

## Abstract

**Objective:**

Lesbian couples must resort to adoption or donated semen to achieve parenthood, the latter usually involving assisted reproductive technology. The aim of this study is to assess homosexual women’s knowledge about assisted reproductive techniques, the importance of perceived genetic and gestational relationships for their future mother-child bond, as well as their reproductive plans.

**Methods:**

This is an observational study based on an anonymous survey disseminated online in several countries on different continents, addressed to homosexual women.

**Results:**

From the 549 participants, most reported being well informed about reproductive options including assisted reproductive technology. The majority want to be a mother as part of a couple, mainly through assisted reproduction or step adoption of their partner’s child. The importance of a genetic or gestational relationships with their future child varies greatly between women. Among the sampled women, pregnancy was believed to have a slightly greater impact on the future mother-child connection compared to genetics.

**Conclusions:**

Homosexual women are well informed about the assisted reproductive technology treatments. The majority considers it important to become a mother as a couple, mainly through assisted reproduction or step adoption of their partner’s child. The importance given to gestation or genetic mother-child relationships varies greatly between women, and it seems they believe pregnancy may have a slightly greater impact on the future mother-child connection compared to genetics.

## INTRODUCTION

Many countries have progressively offered adoption and assisted reproductive treatment (ART) to homosexual couples (Calhaz-Jorge *et al.,* 2020; Brandão, 2022). To undergo reproductive treatment, male couples need an oocyte donor and a surrogate, while female couples need donated semen (Kim, 2017; Mackenzie *et al.,* 2020). For lesbian couples, both intrauterine insemination (IUI) and in vitro fertilization (IVF) with donated sperm are viable options. The former is simpler, cheaper, requires less medication and is associated with higher risks; the later, however, has higher rates of success and allows the creation of surplus embryos which can be used in future treatments in case of treatment failure or desire for a second child (Bardet *et al.,* 2022).

During the last decade, a new method has emerged allowing both members of lesbian couples to be biological mothers of the same child – the ROPA (in Spanish: Recepción de Ovocitos de PAreja, in English: Reception of Oocytes from PArtner), in which the oocytes of one partner (“donor” or “genetic mother”) are fertilized with donated sperm and the resulting embryo is transferred to the other partner’s uterus (“recipient” or “gestational mother”) (Bodri *et al.,* 2018; Brandão *et al.,* 2022a). This method allows both members of lesbian couples to have an active role in procreation (Marina *et al.,* 2010; Brandão *et al.,* 2022d). Recent studies have shown similar outcomes between the ROPA method and one-way IVF (Brandão *et al.,* 2022a).

A growing demand for reproductive treatments for same-sex couples has been observed during the last decade (Brandão & Garrido, 2022). Most lesbian couples go to a fertility clinic in search of a sperm donor (Bokek-Cohen, 2022). Although the outcomes of treatments are not influenced by sexual orientation, recent studies show that a fertility disorder is discovered during medical work-up in 40% of the couples (Soares *et al.,* 2019; Brandão *et al.,* 2022b). Therefore, more than half of lesbian couples require IVF instead of IUI. Interestingly, one study reported that more than 75% of lesbian couples chose a “one parent only” reproductive treatment, which means that only one quarter of the patients chose to be both actively involved, either by undergoing independent concomitant treatments or ROPA (Carpinello et al., 2016; Brandão *et al.,* 2022b).

The impact of biological ties on mother-child bonding in a lesbian couple is unclear. Although some studies suggest mother-child attachment is similar between both mothers, others report that unequal power dynamics derived from a genetic or gestational relationship may lead to interpartner jealousy (Rudd, 2005; Goldberg *et al.,* 2008; Pelka, 2009). The importance of biological ties - gestational or genetic - to these women is far from well understood (Reimann, 1997).

Minimal information exists regarding the knowledge homosexual women have concerning ART and what factors drive their family planning (Brandão *et al.*, 2022c). The purpose of this work is to assess homosexual women’s knowledge about ART, to understand the importance they assign to a gestational and genetic relationship with their future child, to understand how they feel these relationships may impact their future mother-child connection and establish what their future reproductive plans are.

## MATERIAL AND METHODS

### Procedures

This was an observational study based on an anonymous survey applied to individuals who identify themselves as female and homosexual. The study was conducted between May and November 2021.

The links for the surveys were disseminated via social networks, mainly via LGBTIQ+ associations, along with a short message of invitation and presentation of the study.

### Participants

Inclusion criteria were females who identify themselves as homosexual/lesbian. Exclusion criteria were transgender or non-binary individuals, individuals who could not read any of the languages in which the survey was available, patients who had already participated or who refused to answer the survey.

### Survey instrument

At the beginning of the surveys, the inclusion and exclusion criteria were presented. Participants were asked to complete the survey only if they met all the criteria. They were also asked to give their informed consent to participate.

The survey was originally written in English (Supplement 1). It was available as a Google® form, divided into 5 sections: personal data, previous knowledge about assisted reproduction techniques, current family, importance of genetics and pregnancy and future reproductive plans. The survey was translated to French, German, Italian, Latvian, Polish, Portuguese, Romanian and Spanish by native or proficient speakers. The translated versions were re-checked by a second proficient speaker to assure its accurate translation.

### Statistical analysis

The answers were compiled in a SPSS® database for statistical analysis. Means and proportions were calculated for continuous and categorical variables, respectively. Most answers to the outcome questions were in a Likert scale format. They are presented in categories, but for statistical purposes, these answers were analyzed as a continuous score. T-test or Mann-Whitney U test were used to compare parametric or non-parametric variables respectively, after assessing normality by visual appreciation of their distribution. Missing data were excluded.

The outcomes were divided in 3 mam groups: previous knowledge on assisted reproductive treatments, the importance given to genetics and pregnancy and future reproductive plans.

## RESULTS

**Figure 1:**
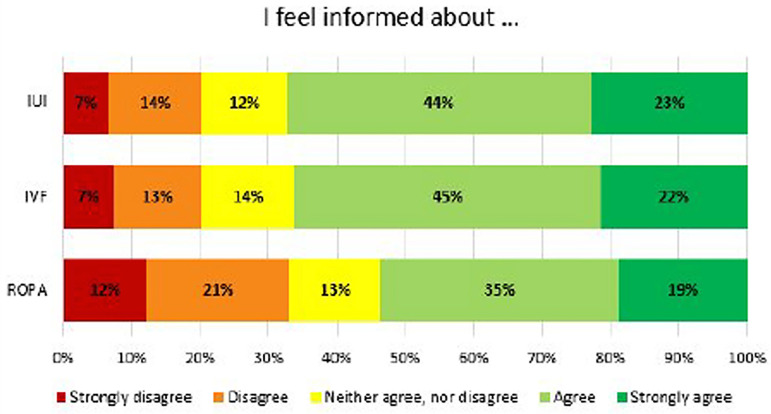
Level of knowledge about assisted reproduction techniques - Answers to the question “To what extent do you agree with the following statements? I feel informed about ...”

A total of 549 responses were obtained. The mean age was 29 years. Most of the participants were from Europe or Latin America. The most represented of the 49 countries were Brazil, Portugal, Spain, Latvia, Romania, UK, USA and Ireland. Most of the participants were in a relationship at the time of the survey, had no children and had some degree of higher education (university graduation, master or PhD). More than 85% had no children, the remaining had had children mainly by IUI/IVF or spontaneous pregnancy (Table 1). More than half of the participants felt informed or strongly informed about IUI and IVF. This percentage was slightly lower for the ROPA method (Figure 1).

**Table 1:** Description of the sample: age, origin, level of studies, current relationship status, number of children and reproductive background.

Description	Results
Age years ( Mean (95% CI))	29.1 (28.0 – 30.2)
Origin (%) Africa Asia Europe Latin America Oceania USA/Canada	0.4% 2.2% 48.1% 45.2% 0.4% 3.8%
Countries (by number of answers):	Brazil, Portugal, Spain, Latvia, Romania, UK, USA, Ireland, Chile, Finland, India, Austria, Belgium, Argentina, Bolivia, El Salvador, Germany, Albania, Bosnia and Herzegovinia, Australia, Angola, Canada, Cape Verde, Colombia, Croatia, Cuba, Czech Republic, Denmark, Ecuador, Estonia, France, Iceland, Italy, Lithuania, Malasia, Mexico, Netherlands, Norway, Peru, Philipines, Poland, Russia, Slovakia, Slovenia, Switzerland, Thailand, Turkey, Uruguay, Venezuela.
Level of studies (%) Elementary school High school Graduation Master PhD	0.7% 16.9% 57.6% 23.3% 1.5%
Current Relationship (%) No Yes	26.8% 73.2%
Number of children (%) 0 1 2 3	86.5% 11.5% 1.3% 0.7%
Reproductive background (%) Previous spontaneous pregnancy Previous adoption Previous stepparent adoption Previous IUI/IVF Previous ROPA	5.1% 0% 1.5% 9.1% 1.5%

– Confidence interval, IUI – Intrauterine insemination, IVF – in vitro fertilization, ROPA – Reception of oocytes from partner, UK – United Kingdom, USA – United States of America.

IUI – Intrauterine insemination

IVF – In vitro fertilization

ROPA – Reception of Oocytes from Partner.

**Figure 2:**
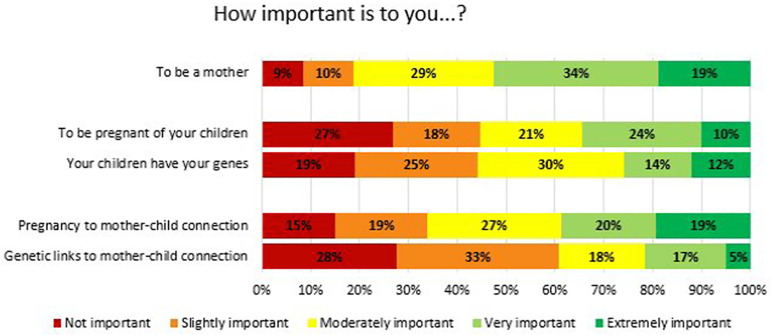
Importance of becoming mother, pregnancy, and genetics - Answers to the question “How important is it to you...?”.

Only 19% of the participants stated that being a mother is not or is slightly important. Regarding the importance of pregnancy and sharing genes with their children, opinions were diverse among the respondents. Studying the variable as a continuous scale from 1 to 5, there were no differences in the scores attributed to both factors (p=0.84). Likewise, the importance given to both pregnancy and genetics to mother-child connection was also variable among the sample studied. Interestingly, when doing the same analysis on a continuous scale, participants tended to attach more importance to pregnancy compared to genetic ties for mother-child bonding (*p*<0.01) (Figure 2).

**Figure 3:**
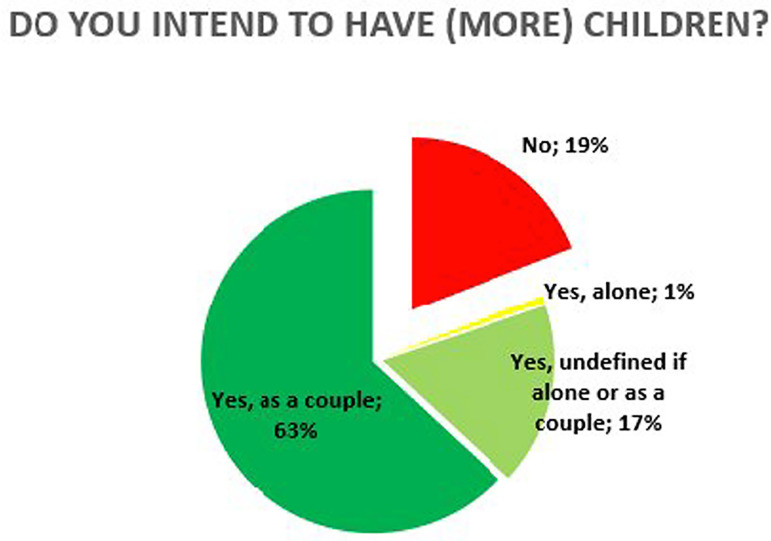
Intention to have more children. Answer to the question “do you intend to have more children?”.

**Figure 4:**
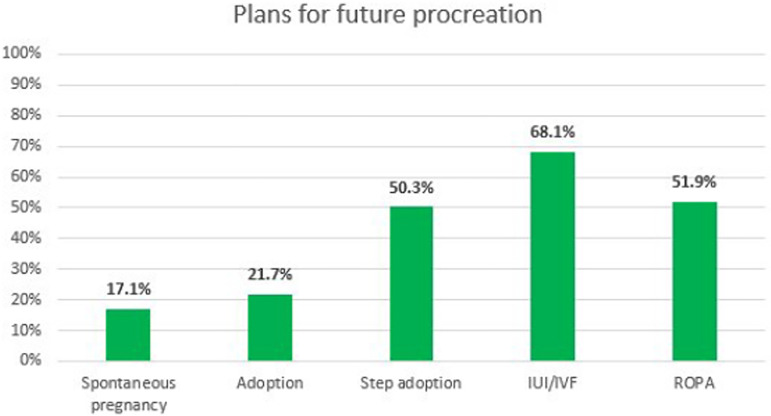
Plans for future procreation.

In the future, only 19% did not consider having children and a large majority planned to do it as a couple (Figure 3). The most reported methods as plans for future procreation were the assisted reproduction techniques – IUI, IVF and ROPA. Half of the respondents would also step adopt a child from their partner. In a smaller percentage came adoption and spontaneous pregnancy (Figure 4).

IUI – Intrauterine insemination

IVF – In vitro fertilization

ROPA – Reception of Oocytes from Partner.

## DISCUSSION

Little is known about how well-informed homosexual women are about the various assisted reproductive treatments. Assisted reproduction had traditionally been largely for heterosexual couples, including associated marketing and patient information provided by fertility clinics (Corbett *et al.*, 2013; Mamo, 2013; Gregg, 2018; Kreines *et al.,* 2018). Interestingly, however, a previous study found that most patients who come to a fertility consultation already have in mind the type of treatment they wish to have, although they often adopt a differing treatment approach following medical advice (Brandão *et al.,* 2022b). In our study, 67% of the participants agree or strongly agree they feel informed about IUI and IVF. This percentage is lower (54%) for the ROPA method. These results suggest that homosexual women, in general, feel informed about methods by which they could achieve biological parenthood, including ART.

In our study, only 19% of women say that becoming a mother is of little or no importance at all. This finding aligns with previous literature; there is a high and increasing number of homosexual women who wish to be a mother (Bos et al., 2003; Turcan *et al.,* 2020).

There are few studies to date on the reproductive intentions of homosexual women, and the available studies are mostly of women already engaging with ART (Carpmello *et al.,* 2016; Brandão *et al.,* 2022b). What guides lesbian couples in their reproductive preferences remains unclear (Brandão *et al.,* 2022c). We addressed the importance of gestational and genetic relationships to understand whether these could be determining factors. Individual opinions regarding these matters were diverse regarding the importance of personal gestational or genetic mother-child relationships, and in beliefs regarding the potential impact of these relationships on future mother-child connection.

No differences were observed in the importance given to pregnancy and genetics in the generation of their own child. However, women assigned slightly more importance to pregnancy regarding future mother-child, compared to genetics, although this difference was small. Literature is conflicting in this respect, some studies pointing to different connections of children with their mothers, while others point to a fairly balanced family situation (Reimann, 1997; Goldberg *et al.,* 2008; Pelka, 2009; Golombok & Badger, 2010).

Regarding reproductive plans in the future, most state they wish to have children and the most reported methods were the assisted reproductive techniques - 68% state they would consider IUI or IVF, 52% consider ROPA. Half of the respondents consider step-adopting the biological child of their partner. Adoption and spontaneous pregnancy (with a friend or acquaintance) are plans of only a minority. Our data suggest that homosexual women value biological motherhood, as previous studies have indicated, although this is not yet clear and obviously varies greatly from patient to patient (Di Nucci, 2016; Voultsos *et al.,* 2019; Costa *et al.,* 2020).

One of the main limitations of this study is the fact that the questionnaire is not validated. The online application and dissemination of the questionnaire does not allow and interface between the researchers and the participants, which means that the inclusion criteria cannot be confirmed. Furthermore, given that an electronic tool was used for data collection, the group of participants comprises a cohort with both internet access and a degree of digital literacy. Additionally, it is important to interpret these results, particularly self-reported knowledge, in the context of the significantly above average background educational attainment of the observed cohort.

Although the study was carried out at a global level and several countries were represented, the vast majority came from countries with high or very high human development indexes, making it impossible to extrapolate the results to the reality of less developed countries, where access to ART is significantly reduced. In addition, there may be a response bias, since we could not find out the proportion of people receiving and seeing the survey who responded.

## CONCLUSION

Homosexual women seem to be well informed about the assisted reproductive technology treatments. The majority of the sample studied considers it important to become a mother and plan to do so as a couple, mainly through assisted reproduction or step adoption of their partner’s child. The importance given to gestation or genetic mother-child relationships varies greatly between women, and it seems they believe pregnancy may have a slightly greater impact on the future mother-child connection compared to genetics.

## Supplement 1 3A - Female gay couples reproductive options

### INTRODUCTION

This inquiry is part of a study about female gay couple’s reproduction.

This inquiry has 5 groups of questions and it’s expected to take less than 2 minutes to complete.

It’s anonymous and your participation is voluntary.

Data will be stored for further analysis and the results may be presented in scientific meetings and/or published in scientific journals or other social media.

By completing this inquiry you are accepting yours answers will be processed according to previously exposed.

**Criteria to participate in this study:** (If you do not fulfill **ALL** of these criteria, please do not answer)

1. You WERE BORN (BIOLOGICAL GENDER) FEMALE
2. You have NOT UNDERGONE ANY TRANSGENDER PROCES,
3. You consider yourself GAY or predominantly ATTRACTED TO WOMEN.

### CONSENT

Do you accept the collection, storage and processing of your answers as well as the publication of the results of this research according to previously exposed in the introduction? **(If you accept, please select “yes”) Yes □**

### CONCEPTS

- **Artificial or intrauterine insemination:** insemination of sperm into the uterus
- ***in vitro* fertilization (IVF):** retrieval of oocytes (ova), fertilization with sperm in laboratory and transfer of an embryo to the uterus
- **Shared IVF** (also known as ROPA): a type of IVF for female couples, in which one woman provides the oocytes (genetic mother) and the other receives the embryo / gets pregnant (gestational mother)

### 1 – PERSONAL DATA

**1a -Age:** ___

**1b** - **Nationality:** ___

**1c – Country of residence:** ___

**1d – Gender with which I identify myself:**

□ Female
□ Without partner
□ Male

**1e – Current relationship:**

□ With partner
□ Non Binary

**1e – Level of education:**

□ No studies
□ Graduation (university)
□ Primary/elementary school
□ Master degree
□ High school
□ PhD

### 2 – INFORMATION

**How INFORMED are you concerning the following techniques?** (Please choose the correct answer :)

**2a – Intrauterine (or artificial) insemination**

1. -□ Uninformed
2. -□ Little informed
3. -□ Moderately informed
4. -□ well Informed
5. -□ very well informed

**2b – *in vitro* fertilization (IVF)**

1. -□ Uninformed
2. -□ Little informed
3. -□ Moderately informed
4. -□ well Informed
5. -□ very well informed

**2c – Shared IVF (ROPA)**

1. -□ Uninformed
2. -□ Little informed
3. -□ Moderately informed
4. -□ well Informed
5. -□ very well informed

### 3 – FAMILY

**3a – Do you have children?:**

□ No
□ Yes (if “yes”, how many: ___)

**3b – Which method(s) have you ALREADY USED to have children?:** (Please choose the correct answer(s) :)

□ Spontaneous pregnancy (previous or current male partner)
□ Spontaneous pregnancy (a friend or an acquaintance)
□ Adoption
□ Adoption of partner’s children

## References

[r1] Bardet L, Excoffier JB, Salaun-Penquer N, Ortala M, Pasquier M, Mathieu d’Argent E, Massin N (2022). Comparison of predictive models for cumulative live birth rate
after treatment with ART. Reprod Biomed Online.

[r2] Bodri D, Nair S, Gill A, Lamanna G, Rahmati M, Arian-Schad M, Smith V, Linara E, Wang J, Macklon N, Ahuja KK (2018). Shared motherhood IVF: high delivery rates in a large study of
treatments for lesbian couples using partner-donated eggs. Reprod Biomed Online.

[r3] Bokek-Cohen Y (2022). Sperm donors versus long-term mates: a comparison of preferences
of heterosexual and lesbian women. Hum Fertil (Camb).

[r4] Bos HM, van Balen F, van den Boom DC (2003). Planned lesbian families: their desire and motivation to have
children. Hum Reprod.

[r5] Brandão P (2022). European policies on same-sex relationships, adoption and
assisted reproduction. Int J Reprod Contraception Obstet Gynecol.

[r6] Brandão P, Garrido N (2022). Commercial Surrogacy: An Overview. Rev Bras Ginecol Obstet.

[r7] Brandão P, Ceschin N, Cruz F, Sousa-Santos R, Reis-Soares S, Bellver J (2022a). Similar reproductive outcomes between lesbian-shared IVF (ROPA)
and IVF with autologous oocytes. J Assist Reprod Genet.

[r8] Brandão P, Ceschm N, Gómez VH (2022b). The Pathway of Female Couples in a Fertility
Clinic. Rev Bras Ginecol Obstet.

[r9] Brandão P, Ceschin N, Sandvik B, Paolelli S, Doblinger J, Reis-Soares S, Sousa-Santos R, Bellver J (2022c). Female couples undergoing assisted reproduction - choices and the
importance of pregnancy and genetic. Braz J Reprod Assist.

[r10] Brandão P, de Pinho A, Ceschin N, Sousa-Santos R, Reis-Soares S, Bellver J (2022d). ROPA – Lesbian shared in vitro fertilization – Ethical
aspects. Eur J Obstet Gynecol Reprod Biol.

[r11] Calhaz-Jorge C, De Geyter CH, Kupka MS, Wyns C, Mocanu E, Motrenko T, Scaravelli G, Smeenk J, Vidakovic S, Goossens V (2020). Survey on ART and IUI: legislation, regulation, funding and
registries in European countries: The European IVF-monitoring Consortium
(EIM) for the European Society of Human Reproduction and Embryology
(ESHRE). Hum Reprod Open.

[r12] Carpinello OJ, Jacob MC, Nulsen J, Benadiva C (2016). Utilization of fertility treatment and reproductive choices by
lesbian couples. Fertil Steril.

[r13] Corbett SL, Frecker HM, Shapiro HM, Yudin MH (2013). Access to fertility services for lesbian women in
Canada. Fertil Steril.

[r14] Costa PA, Tasker F, Carneiro FA, Pereira H, Leal I (2020). Reactions from family of origin to the disclosure of lesbian
motherhood via donor insemination. J Lesbian Stud.

[r15] Di Nucci E (2016). IVF, same-sex couples and the value of biological
ties. J Med Ethics.

[r16] Goldberg AE, Downing JB, Sauck CC (2008). Perceptions of children’s parental preferences in lesbian
two-mother households. J Marriage Fam.

[r17] Golombok S, Badger S (2010). Children raised in mother-headed families from infancy: a
follow-up of children of lesbian and single heterosexual mothers, at early
adulthood. Hum Reprod.

[r18] Gregg I (2018). The Health Care Experiences of Lesbian Women Becoming
Mothers. Nurs Womens Health.

[r19] Kim HH (2017). Family Building by Same-Sex Male Couples via Gestational
Surrogacy. Semin Reprod Med.

[r20] Kreines FM, Farr A, Chervenak FA, Grunebaum A (2018). Quality of web-based family-building information for LGBTQ
individuals. Eur J Contracept Reprod Heal Care.

[r21] Mackenzie SC, Wickins-Drazilova D, Wickins J (2020). The ethics of fertility treatment for same-sex male couples:
Considerations for a modern fertility clinic. Eur J Obstet Gynecol Reprod Biol.

[r22] Mamo L (2013). Queering the fertility clinic. J Med Humanit.

[r23] Marina S, Marina D, Marina F, Fosas N, Galiana N, Jové I (2010). Sharing motherhood: biological lesbian co-mothers, a new IVF
indication. Hum Reprod.

[r24] Pelka S (2009). Sharing motherhood: maternal jealousy among lesbian
co-mothers. J Homosex.

[r25] Reimann R (1997). Does Biology Matter?: Lesbian Couples’ Transition to Parenthood
and Their Division of Labor. Qual Sociol.

[r26] Rudd E (2005). Inside Lesbian Families: The Family of Woman: Lesbian Mothers,
Their Children, and the Undoing of Gender by Maureen Sullivan University of
California Press, 2004, 312 pages. Contexts.

[r27] Soares SR, Cruz M, Vergara V, Requena A, García-Velas-co JA (2019). Donor IUI is equally effective for heterosexual couples, single
women and lesbians, but autologous IUI does worse. Hum Reprod.

[r28] Turcan P, Prochazka M, Pokorny P, Kvintova J, Sigmund M, Juraskova ES (2020). Desire for Parenthood and Associated Trends in Czech Lesbian
Women. Sex Med.

[r29] Voultsos P, Zymvragou CE, Raikos N, Spiliopoulou CC (2019). Lesbians’ experiences and attitudes towards parenthood in
Greece. Cult Health Sex.

